# Marital Religious Homogamy and Dimensions of Well-Being in Later Life: Evidence From the United States

**DOI:** 10.1093/geroni/igaa057.981

**Published:** 2020-12-16

**Authors:** Laura Upenieks, Markus Schafer, Jeremy Uecker

**Affiliations:** 1 University of Texas at San Antonio, San Antonio, Texas, United States; 2 University of Toronto, Toronto, Ontario, Canada; 3 Baylor University, Waco, Texas, United States

## Abstract

Past research points to the importance of couple-level religious similarity for multiple dimensions of older adults’ partnership quality and stability, but we have a limited understanding of whether religious homogamy matters for the well-being of seniors. This study uses dyadic data from the National Social Life, Health, and Aging Project (NSHAP), a representative sample of 953 individuals ages 62–91 plus their marital or cohabiting partners. Using actor-partner interdependence models in the general structural equation model framework (GSEM), we find that religious attendance homogamy is beneficial for the physical health of men and the mental health and self-reported happiness of women. There were no associations between religious homogamy for religious importance detected. Taken together, our results attest to the ongoing importance of religious similarity—service attendance, in particular—for mental and physical well-being in later life. Future research is needed to more fully examine which mechanisms account for these patterns.

